# Marked differences in foraging area use and susceptibility to predation between two closely-related tropical seabirds

**DOI:** 10.1007/s00442-023-05459-x

**Published:** 2023-10-10

**Authors:** Annette L. Fayet, Cheryl Sanchez, Jennifer Appoo, Jessica Constance, Gemma Clucas, Lindsay A. Turnbull, Nancy Bunbury

**Affiliations:** 1 Norwegian Institute for Nature Research (NINA), Trondheim, Norway; 2 Department of Biology, University of Oxford, Oxford, UK; 3 Seychelles Islands Foundation, Victoria, Seychelles; 4 Department of Biology, University of Pisa, Pisa, Italy; 5 UMR ENTROPIE, Université de La Réunion, Saint-Denis, La Réunion France; 6 Cornell Lab of Ornithology, Cornell University, Ithaca, USA; 7 Centre for Ecology and Conservation, University of Exeter, Penryn, UK

**Keywords:** Invasive species, Niche partitioning, Spatial segregation, Seychelles, Sympatry

## Abstract

Ecological theory predicts that closely-related species must occupy different niches to coexist. How marine top predators achieve this during breeding, when they often gather in large multi-species colonies and are constrained to central-place foraging, has been mostly studied in productive temperate and polar oceans with abundant resources, but less so in poorer, tropical waters. Here, we track the foraging movements of two closely-related sympatric seabirds—the white-tailed and red-tailed tropicbirds *Phaethon lepturus* and *P. rubricauda*—breeding on Aldabra Atoll, Seychelles, to investigate potential mechanisms of niche segregation and shed light on their contrasting population trends. Combining data from GPS, immersion, depth and accelerometry loggers, we show that the two species have similar behaviour at sea, but are completely segregated spatially, with red-tailed tropicbirds flying further to feed and using different feeding areas than white-tailed tropicbirds. Using nest-based camera traps, we show that low breeding success of both species—which likely drives observed population declines—is caused by high nest predation. However, the two species are targeted by different predators, with native avian predators mainly targeting red-tailed tropicbird nests, and invasive rats raiding white-tailed tropicbird nests when they leave their eggs unattended. Our findings provide new insight into the foraging ecology of tropicbirds and have important conservation implications. The extensive range and spatial segregation highlight the importance of considering large-scale protection of waters around tropical seabird colonies, while the high level of nest predation provides evidence in support of rat eradication and investigating potential nest protection from native avian predators.

**Supplementary Information:**

The online version contains supplementary material available at 10.1007/s00442-023-05459-x.

## Introduction

The mechanisms of coexistence between closely related species are paramount to understanding adaptation and speciation within ecological communities. A key assumption of traditional ecological theory is that species will occupy different niches (Gauze 1934), while neutral theory challenges this principle (Hubbell 2001). In the marine environment, examples of apparently similar species coexisting are particularly common. For example, top predators, such as seabirds and marine mammals, often feed on small fish and regroup in large multi-species colonies to breed, potentially leading to intense competition (Lewis et al. 2001). Mechanisms that reduce interspecific competition are therefore likely to be important. With many marine top predators currently in decline (McCauley et al. 2015; Dias et al. 2019), these questions are increasingly relevant.

There have been several detailed studies of the coexistence of marine predators in productive environments, such as those found in temperate and polar regions (Rosenzweig 1995; Pigot et al. 2016). At these latitudes, some co-nesting seabirds appear to have similar feeding niches (Dehnhard et al. 2020; Planque et al. 2021), while others show niche differentiation through a range of mechanisms including: feeding in different areas (Hamilton et al. 2019), at different depths (Hoskins et al. 2017) or at different times (Clewlow et al. 2019). In addition, species often specialise on different kinds or sizes of prey (Barger and Kitaysky 2012).

However, it is less well understood how closely-related, sympatric species coexist in tropical waters, where food is generally less available and more patchily distributed. Several studies have used diet sampling or stable isotopes to compare diet, trophic levels or overlap in foraging habitats (Kojadinovic et al. 2008; Catry et al. 2008, 2009; Pontón-Cevallos et al. 2017). Alone, however, these methods cannot reveal more subtle differences in spatial distribution. Combining multiple approaches, such as tracking foraging movements alongside diet sampling, could provide a more complete picture but remains limited (Kiszka et al. 2011; Mott et al. 2017).

Here, we investigate how two closely-related tropical seabirds, the white-tailed and the red-tailed tropicbirds *Phaethon lepturus* and *P. rubricauda*, coexist in a breeding colony at Aldabra Atoll in Seychelles. While the two tropicbird species differ in size and breeding phenology (see Methods), they have similar breeding habits and diet (Diamond 1975; Catry et al. 2009; Le Corre et al. 2003), making them ideal candidates to study coexistence mechanisms in more detail. Currently, we know very little about their spatial ecology during breeding, with a single published GPS tracking study of white-tailed tropicbirds, carried out in the Atlantic Ocean (Campos et al. 2018).

A better understanding of tropicbird foraging ecology at Aldabra is also important for their conservation, e.g. by revealing the extent to which the Marine Protected Area network covers their foraging grounds. Aldabra is a biodiversity hotspot and a key breeding colony for many seabird species in the Western Indian Ocean, including for red-tailed tropicbirds, with potentially almost 30% of the Indian Ocean’s population breeding on the island (Diamond 1971; Schreiber and Schreiber 2020). Population monitoring at Aldabra has revealed that the number of red-tailed tropicbird nests has strongly declined since 2010 (2010–2019: − 75.9%, Seychelles Islands Foundation (SIF), unpublished data). In contrast, the number of white-tailed tropicbird nests appears more stable (2010–2019: + 1.8%), but their breeding success is poor (Burt et al. 2021), so their population might be affected in the future. Understanding the foraging ecology of both species may help to understand why. Indeed, foraging strategies and demographic traits can be linked (Morales et al. 2010). For instance, low food availability can force animals to forage further, with negative effects on breeding success through poor offspring condition (Fayet et al. 2021) or increased predation risk (Brickle et al. 2000). Foraging strategies can also be reflected in adult survival, e.g. through exposure to threats at the foraging grounds (Genovart et al. 2018). On Aldabra, the contrasting trends of the two tropicbird species could be due to one species having less access to food (e.g. by having to travel further to reach prey, or by foraging at less productive grounds). They may also experience different predation risks. Aldabra is home to a population of introduced black rats (*Rattus rattus*) and to several potential avian predators. White-tailed and red-tailed tropicbirds may be targeted differently by these predators; alternatively, differences in their foraging strategies—such as how long they leave the nest unattended—may affect their propensity for predation (Blight et al. 1999).

To address these knowledge gaps and obtain a comprehensive picture of the foraging ecology of red-tailed and white-tailed tropicbirds breeding on Aldabra, we combined multiple approaches. To determine their foraging distribution, environmental preferences, and potential spatial segregation, we tracked both species during incubation and chick-rearing with GPS devices. To investigate their foraging behaviour in more detail, we combined the GPS data with immersion and depth/accelerometry loggers on a subsample of birds. In parallel, to investigate diet, we analysed regurgitates and used DNA metabarcoding techniques on faecal samples. Finally, to assess the impact of nest predation and potential links with the birds’ foraging behaviour, we monitored nests with camera traps.

## Methods

### Study site and species

Data collection took place in January-March 2018 and 2019 on Aldabra Atoll, Seychelles (− 9.42°N, 46.34°W). Aldabra is a large, raised coral atoll in the Western Indian Ocean managed by SIF.

White-tailed tropicbirds inhabit all three tropical oceans, while red-tailed tropicbirds only occur in the Indian and Pacific Oceans. Both species lay a single egg and have biparental care. On Aldabra, red-tailed tropicbirds mainly breed between October and April while white-tailed tropicbirds breed year-round (Prys-Jones and Peet 1980). Red-tailed tropicbirds nest on the ground under vegetation cover, while white-tailed tropicbirds favour rocky crevices (Diamond 1975). Likely because of invasive terrestrial predators on the main island (rats and cats, the latter occurring only on one of the four main islands forming the atoll), both species mainly nest on islets in the lagoon. Nevertheless, rats are able to reach some islets. At sea, both species are shallow plunge divers (Schreiber and Clapp 1987) and feed mostly solitarily (Jaquemet et al. 2004).

### Data collection

Nests were found on islets in three areas in the western lagoon (La Gigi, Point Tanguin, and Gionnet). In 2019, birds (14 white-tailed tropicbirds, average body mass 325 g, 24 red-tailed tropicbirds, average body mass 733 g) were caught at the nest by hand and fitted with a miniature GPS device attached to the central tail feathers using thin strips of marine cloth tape (back attachments were trialled the previous year but all devices fell off within days). All were incubating an egg except four white-tailed tropicbirds and six red-tailed tropicbirds which were rearing small chicks. Nine white-tailed tropicbirds were fitted with a PathTrack Nanofix remote-download GPS logger (4g, 1.1–1.3% body mass), and 17 red-tailed tropicbirds were fitted with a CatLog Gen2 GPS logger (10g, 1.1–1.6% body mass). Five white-tailed tropicbirds and seven red-tailed tropicbirds were fitted with a TechnoSmart AxyTrek depth/tri-axial accelerometer/GPS logger recording depth and temperature every second and tri-axial acceleration at 25Hz (6g, 1.8–2.2% (white-tailed tropicbirds) or 0.7–0.9% (red-tailed tropicbirds) body mass). All GPS loggers recorded position every 10 min. In addition, the remote-download loggers were configured to emit a UHF signal every 10 min, which the base station (PathTrack) placed in the vicinity of the nests was continually listening for. A subset of birds (ten white-tailed tropicbirds and 17 red-tailed tropicbirds) were also fitted with a geolocator (Migrate Technology C65, 1g, total mass of GPS + GLS 0.8–1.8% (red-tailed) or 1.3–2.6% (white-tailed) body mass) recording maximum light every five minutes and immersion (wet/dry) every six seconds. Bird handling took < 10 min per bird. Birds were recaptured 4–15 days later to remove the device(s). Breast feathers were collected for DNA sexing. Seven birds evaded recapture because their nests failed (causes of nest failure recorded by camera traps: heavy rain killing a newly hatched chick, egg breakage by the adult, rat predation, and two unknown), and one GPS tags failed to record data. Our final dataset therefore comprised tracks from 12 white-tailed tropicbirds (28 trips) and 18 red-tailed tropicbirds (21 trips). Depth loggers recorded 186 and 275 dives from white-tailed and red-tailed tropicbirds, respectively.

Regurgitates and faecal samples were opportunistically collected during handling to investigate diet (14 from white-tailed tropicbirds (eight regurgitated, six faecal), 23 from red-tailed tropicbirds (four regurgitated, 19 faecal)). Faecal samples and fragments of regurgitates were immediately stored in plastic micro-centrifuge tubes filled with 1ml RNAlater (Invitrogen), then frozen at − 18 °C until processed. The samples were later analysed by extracting and sequencing DNA to identify fish prey following the method in Fayet et al. (2021) (details in SI). When possible, the species in regurgitates were identified visually; those samples were used as calibration in the DNA analysis, while the unidentifiable ones were analysed to identify fish prey.

Motion-activated camera traps (Browning Reckon Force Extreme IR) were set up near 62 nests (22 white-tailed, 40 red-tailed, including 26 tracked nests) to record breeding success and potential predation. They were removed after the nest fledged a chick, failed, or at the end of March each year, whichever came sooner. Cameras were deployed on average for 26 ± 15 days (mean ± SD) per nest (range 4–83 days). In total they recorded > 400,000 photos over 1164 nest-days. Photos were analysed manually. Breeding outcome was obtained for 19 white-tailed tropicbirds and 27 red-tailed tropicbirds nests (the other nests were still active when the study ended).

### Behavioural classification analysis

The birds’ behaviour was classified using machine learning models in R (*tidymodels* package, Kuhn and Wickham 2020) (details in SI). Briefly, accelerometer and dive data (n = 10 birds) were assigned to behavioural classes using an unsupervised machine learning approach. Six behaviours were identified: ‘at the nest’, ‘foraging’, ‘sitting on the sea surface during the day’, ‘sitting on the sea surface at night’, and two types of flight which mainly differed in wingbeat frequency but which we grouped together as ‘flight’ for simplicity. The results were then used to label the GPS and light/immersion data collected from the same birds. The labelled data was then used to train a supervised machine learning model to classify behaviour from the rest of the data from the birds tracked with a GPS and/or GPS + geolocator (n = 20). For this step, we compared the performance of Naïve Bayes, Multivariate Adaptive Regression Splines (MARS), Neural Network and Gradient Boosted Trees models, the latter had the highest performance so was retained.

### Spatial data analysis

Trips were defined as the locations between a bird going beyond, and subsequently returning within, a 1-km radius around the nest. Forty-nine trips were identified. We excluded 11 very short trips taken by three incubating white-tailed tropicbirds without a changeover in partners, and described these separately as their function was likely different. To estimate spatial segregation between species, 95% and 50% density kernels were calculated and their overlap measured. For both species, the 95% and 50% home ranges plateaued well before we reached our maximum sample size (Figure S1), demonstrating that we obtained representative utilisation distributions. At each GPS location, depth data were extracted from the GEBCO Gridded Bathymetry Data, and daily values of chlorophyll-A and SST were extracted from the Aqua-MODIS and NASA JPL datasets (resolution 0.04° and 0.01°, respectively) and used to calculate an average over the month preceding the GPS recording. These values were then averaged for each trip.

Linear mixed-effects models were used to test for differences in trip metrics between species and breeding stage (incubation and chick-rearing). Sex was initially included in all models but was not significant in any model, so was removed. When testing for differences in chlorophyll-A, there was a significant interaction between species and stage on chlorophyll-A (χ^2^_1_ = 5.6, p = 0.018), so we analysed each stage separately. In some models, bird identity (as a random effect) explained zero variance and so led to a singular fit. In those cases, we removed the random effect and used a linear model instead. Normality of residuals was checked for all models, and some variables were log or square-root-transformed to meet this assumption.

## Results

### Movements and distribution

**Fig. 1:**
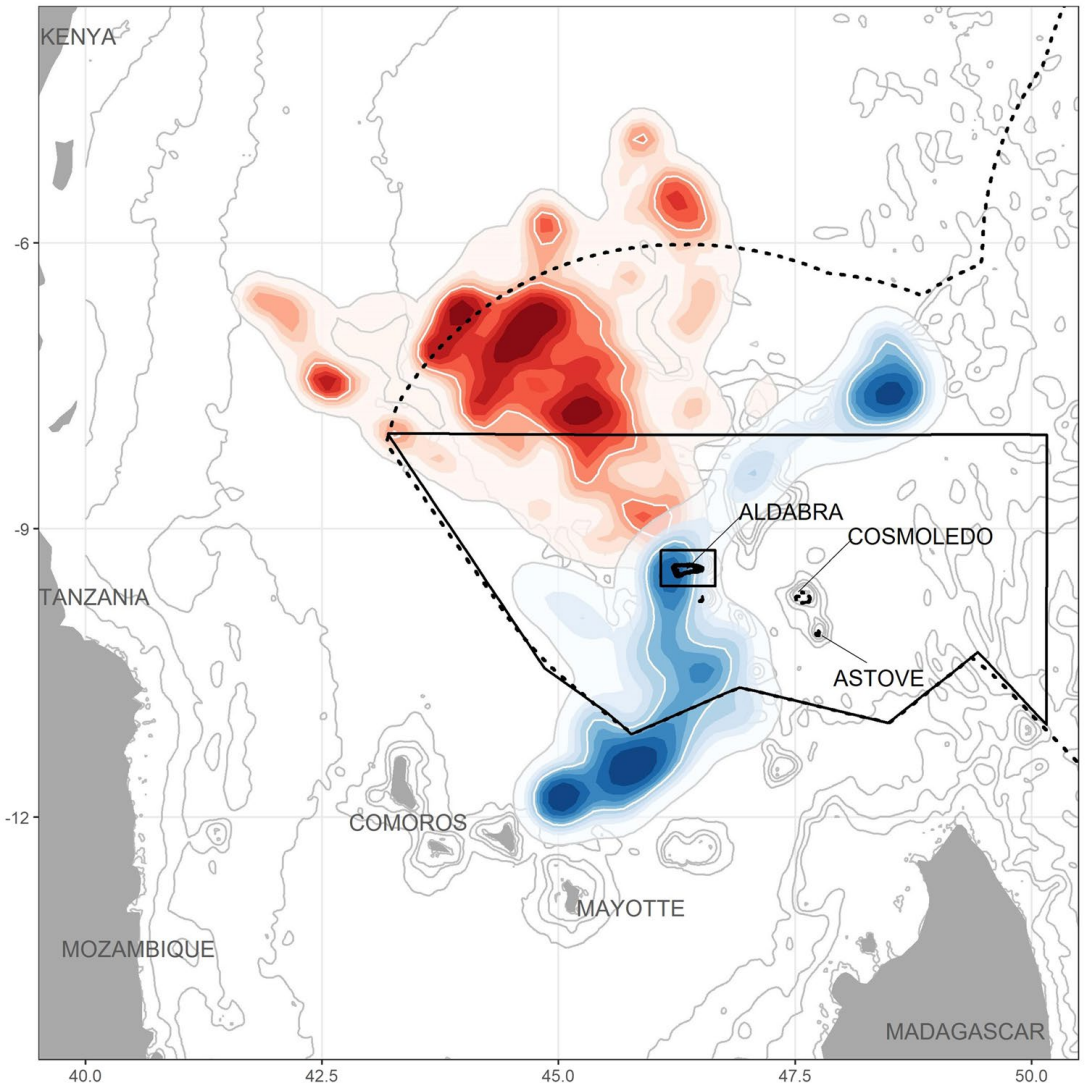
Distribution of red-tailed tropicbirds (red) and white-tailed tropicbirds (blue) during their foraging trips around Aldabra. The different hues represent different contours, from 95% (palest) to 10% (darkest). The core foraging area (50%) is delineated with a white line. The background map shows bathymetry. The dotted black line represents the edge of the Seychelles EEZ, the black lines represent the Aldabra Special Marine Reserve (small rectangle around the island) and the Spatial Marine Planning Area Zone 1. The full tracks are available on Figure S2

Red-tailed tropicbirds used a large area north-west of Aldabra, towards the Somali Basin, while white-tailed tropicbirds went mostly south, towards Mayotte and the Comoros (Fig. 1), and also north-east, to a lesser extent. There was very clear spatial segregation between species, with the distributions at the 95% contour level overlapping by less than 5% (white-tailed tropicbirds: 4.4%, red-tailed tropicbirds: 3.0%). All observed overlap occurred near the colony, while the overlap was zero in the core areas (50% contour). The Bhattacharyya's affinity index, which quantifies the overlap between two utilisation distributions as a single value between 0 (no overlap) and 1 (identical distributions), was 0.09 between the two species.

**Fig. 2:**
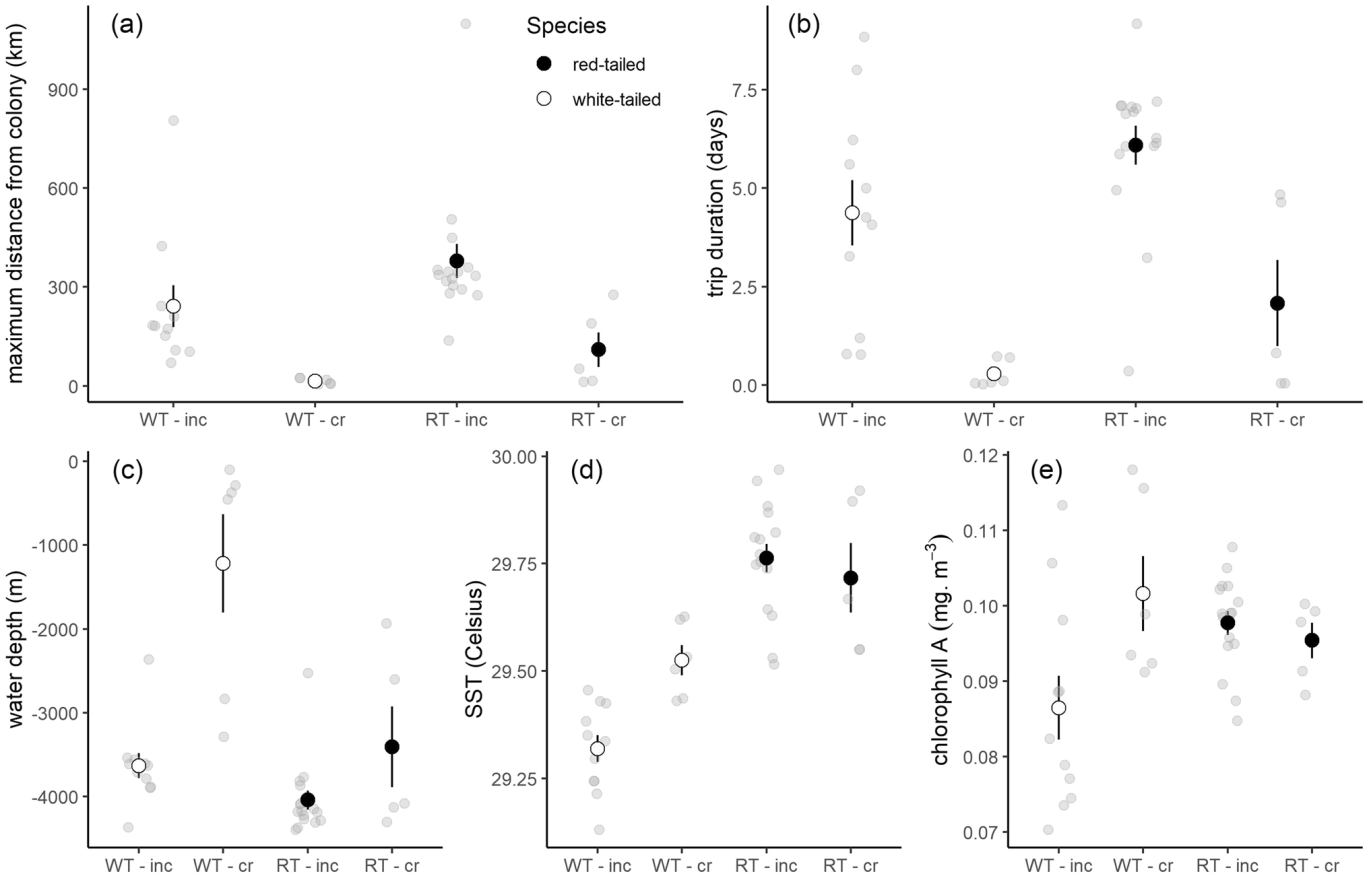
Trip metrics (mean ± SE) from white-tailed tropicbirds (white circles, “WT” label on the x-axis) and red-tailed tropicbirds (black circles, “RT” label) during incubation (“inc” label) and chick-rearing (“cr” label), including maximum distance from the colony (**a**), total trip duration (**b**), water depth (**c**), sea-surface temperature (**d**) and chlorophyll-A (**e**). The grey points represent the average for each trip

The distance and duration of trips differed between species and stages (values and statistics in Table 1). Red-tailed tropicbirds travelled substantially further than white-tailed tropicbirds during both stages (maximum distance from the colony: 379.2 ± 51.7 vs 241.9 ± 63.1 km during incubation, 109.7 ± 52.9 vs 14.7 ± 3.6 km during chick-rearing; Fig. 2a) although we may have underestimated the range of white-tailed tropicbirds during chick-rearing, as all were rearing very young chicks, unlike red-tailed tropicbirds. Trip duration was also substantially greater in red-tailed tropicbirds, lasting an extra two days on average (6.1 ± 0.5 vs 4.4 ± 0.8 days during incubation, 2.1 ± 1.1 vs 0.3 ± 0.1 days during chick-rearing; Fig. 2b). There were also differences between breeding stages, with incubation trips being substantially longer, both in distance and duration, than chick-rearing trips for both species.

**Table 1:** Trip metrics (averaged per trip, mean ± SE) and their differences between species and breeding stage (INC = incubation, CR = chick-rearing) for 21 red-tailed tropicbird trips (INC: n = 16, CR: n = 5) and 17 white-tailed tropicbird trips (INC: n = 11, CR: n = 6)

Trip metric	White-tailed tropicbird	Red-tailed tropicbird	Statistical difference
Movement variables			
Maximum distance from the colony (km)	241.9 ± 63.1 (INC) 14.7 ± 3.6 (CR)	379.2 ± 51.7 (INC) 109.7 ± 52.9 (CR)	Species: F_1,35_ = 15.1, p < 0.001 Stage: F_1,35_ = 39.3, p < 0.001
Trip duration (days)	4.4 ± 0.8 (INC) 0.3 ± 0.1 (CR)	6.1 ± 0.5 (INC) 2.1 ± 1.1 (CR)	Species: F_1,35_ = 10.1, p = 0.003 Stage: F_1,35_ = 27.8, p < 0.001
Total trip distance (km)	829.9 ± 275.6 (INC) 42.2 ± 16.1 (CR)	1150.7 ± 191.0 (INC) 333.1 ± 161.4 (CR)	Species: F_1,35_ = 16.8, p < 0.001 Stage: F_1,35_ = 56.7, p < 0.001
Flight speed (km/h)	16.3 ± 1.1 (mean) 40.3 ± 2.9 (max)	20.8 ± 0.9 (mean) 58.2 ± 2.7 (max)	Species: F_1,36_ = 7.1, p = 0.008 Species: F_1,36_ = 20.1, p < 0.001
Environmental variables			
Chlorophyll (in preceding month) (μg/m^3^)	86.4 ± 4.2 (INC) 101.6 ± 4.9 (CR)	97.7 ± 1.6 (INC) 95.3 ± 2.4 (CR)	Species (INC): F_1,25_ = 8.0, p = 0.009 Species (CR): F_1,11_ = 1.3, p = 0.256
SST (in preceding month) (°C)	29.3 ± 0.03 (INC) 29.5 ± 0.03 (CR)	29.8 ± 0.03 (INC) 29.7 ± 0.08 (CR)	Species: χ^2^_1_ = 36.4, p < 0.001 Stage: χ^2^_1_ = 1.9, p = 0.169
Bathymetry (m)	− 3629 ± 145 (INC) − 1221 ± 586 (CR)	− 4040 ± 111 (INC) − 3406 ± 479 (CR)	Species: χ^2^_1_ = 8.8, p = 0.003 Stage: χ^2^_1_ = 19.5, p < 0.001
Behavioural metrics			
Proportion of flight during daylight (%)	50.2 ± 6.6 (INC) 84.8 ± 4.2 (CR)	47.8 ± 3.6 (INC) 68.2 ± 10.4 (CR)	Species: F_1,35_ = 2.8, p = 0.103 Stage: F_1,35_ = 18.6, p < 0.001
Proportion of foraging during daylight (%)	3.7 ± 0.7 (INC) 4.7 ± 1.6 (CR)	3.3 ± 0.6 (INC) 2.9 ± 1.5 (CR)	Species: χ^2^_1_ = 0.1, p = 0.705 Stage: χ^2^_1_ = 2.4, p = 0.118
Proportion of sitting on the water during daylight (%)	40.3 ± 5.4 (INC) 10.8 ± 4.7 (CR)	44.8 ± 3.1 (INC) 26.8 ± 9.4 (CR)	Species: F_1,35_ = 4.5, p = 0.042 Stages: F_1,35_ = 18.5, p < 0.001
Proportion of flight during night-time (%)	12.4 ± 8.3 (INC) 0 ± 0 (CR)	8.6 ± 3.3 (INC) 1.2 ± 1.1 (CR)	Species: F_1,29_ = 0.5, p = 0.472 Stage: F_1,29_ = 2.9, p = 0.098
Proportion of foraging during night-time (%)	0.8 ± 0.2 (INC) 0 ± 0 (CR)	0.9 ± 0.2 (INC) 0.1 ± 0.1 (CR)	Species: F_1,29_ = 0.5, p = 0.472 Stage: F_1,29_ = 2.9, p = 0.098
Proportion of sitting on the water during night-time (%)	95.5 ± 9.6 (INC) 33.0 ± 20.9 (CR)	96.4 ± 3.7 (INC) 61.6 ± 25.2 (CR)	Species: F_1,29_ = 0.03, p = 0.864 Stage: F_1,29_ = 0.2, p = 0.624
Dive depth (m)	− 0.6 ± 0.07 (mean) − 1.1 ± 0.2 (max)	− 0.6 ± 0.1 (mean) − 1.5 ± 0.2 (max)	Species: F_1,9_ = 0.05, p = 0.824 Species: F_1,9_ = 1.6, p = 0.239
Number of take-offs & landings per day (INC only)	77.7 ± 4.7	76.3 ± 4.5	Species: F_1,16_ = 0.08, p = 0.783
Number of wet bouts (per 24h period, INC only)	23.3 ± 1.6 (day) 1.5 ± 0.2 (night)	16.2 ± 0.6 (day) 2.3 ± 0.1 (night)	Species: F_1,16_ = 25.2, p < 0.001 Species: F_1,16_ = 11.3, p = 0.004
Duration of wet bout (INC only) (hrs)	0.35 ± 0.04 (day) 7.3 ± 0.8 (night)	0.47 ± 0.02 (day) 4.9 ± 0.4 (night)	Species: F_1,16_ = 10.5, p = 0.005 Species: F_1,16_ = 8.7, p = 0.009

The statistical difference column gives results from LMMs testing differences between species and, for some variables, breeding stage

In addition to the foraging trips described above, we recorded 11 short trips by three incubating white-tailed tropicbirds during which the incubating bird left the egg unattended for a short period of time (from half an hour to just over an hour). Such behaviour was also seen regularly on the camera traps (only in white-tailed tropicbirds). These trips were short in range (average maximum distance from the nest 4.1 ± 0.9 km, range 1.1–9.0 km) and duration (49 min, range 29–72 min). One bird took short trips around midday, the other two mostly early in the morning. During the trips, the birds spent most of their time in flight (79.2 ± 8.9%). Based on three trips by one bird fitted with an immersion logger, the bird spent about 15% of the trips wet and did numerous take-offs and landings (on average 12.3 per hour), which could have been feeding but also cleaning or cooling behaviour.

Red-tailed tropicbirds flew faster than white-tailed tropicbirds (average speed: 20.8 ± 0.9 vs 16.3 ± 1.1 km/h, max speed: 58.2 ± 2.7 vs 40.3 ± 2.9 km/h). Environmental conditions encountered at sea varied between species and breeding stages (Table 1). Red-tailed tropicbirds visited substantially deeper waters (hundreds to thousands of meters of difference, “bathymetry” in Table 1, Fig. 2c) and slightly warmer waters (0.2—0.5 °C difference on average, Fig. 2d) than white-tailed tropicbirds. During incubation (but not chick-rearing) they also visited waters with higher chlorophyll-A concentration than white-tailed tropicbirds (97.7 ± 1.6 vs 86.4 ± 4.2 µg/m^3^, Fig. 2e).

### Behaviour

**Fig. 3:**
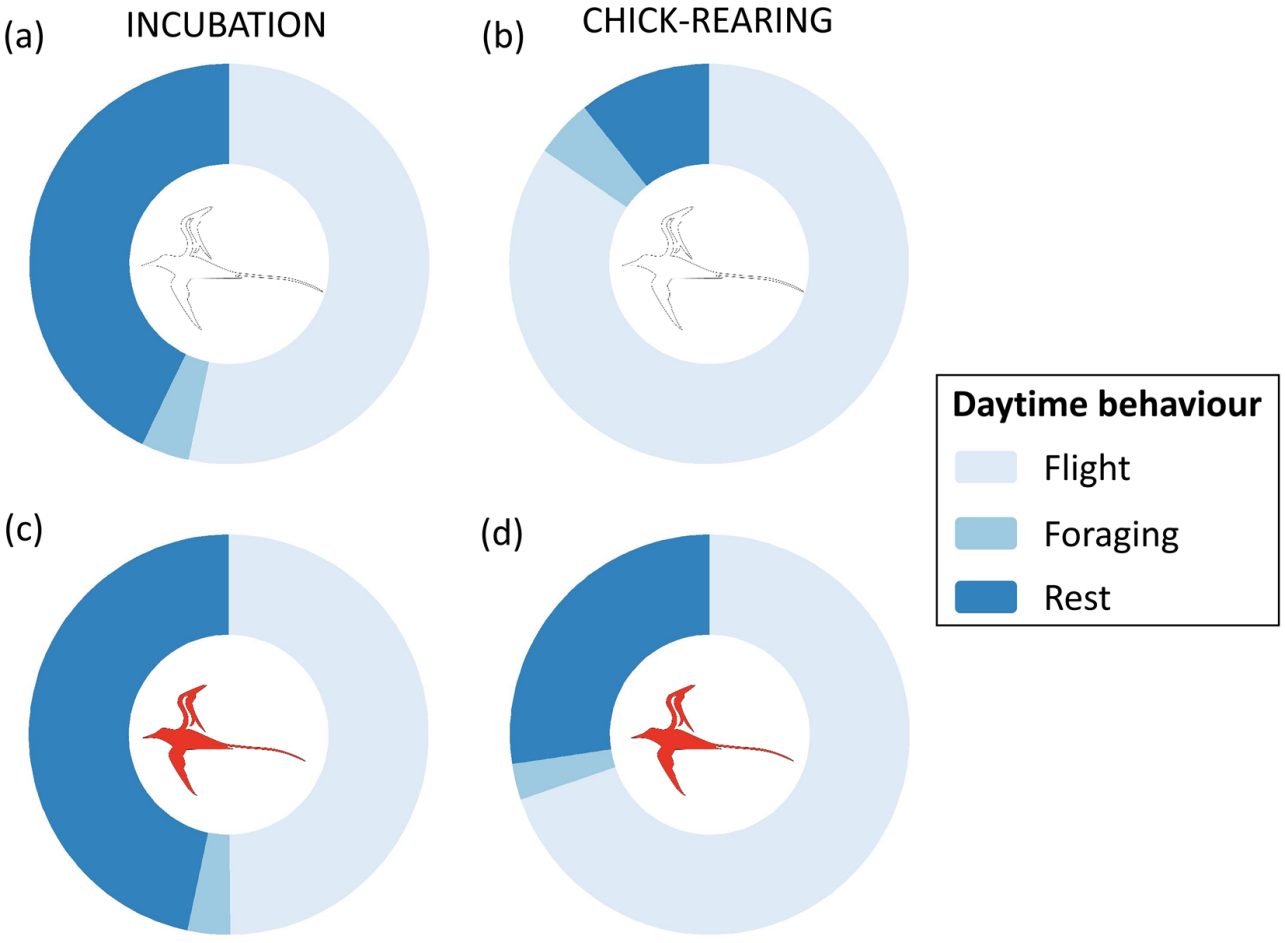
Daytime activity budgets at sea during foraging trips of white-tailed tropicbirds (**a** and **b**) and red-tailed tropicbirds (**c** and **d**) during incubation and chick-rearing. The values shown are means over all trips, exact values are in Table 1

The birds’ activity budgets (proportion of time spent in different behaviours) during daylight hours varied between breeding stages but not species, except for the time spent sitting on the water, which varied with species and breeding stage (values and statistics in Table 1, see Table S6 for absolute durations). During the day, birds spent most of their time in flight (commuting, prey searching; on average between 48% and 85% of their time, depending on stage and species), followed by sitting on the water (between 11 and 45% of their time, depending on stage and species), with < 5% spent foraging (Fig. 3; note: our behavioural classification of foraging relates specifically to dipping in and out of the water, so most prey searching is included in the flight behaviour). At night, birds were mainly sitting on the water (> 95% of the time during incubation, less during chick-rearing). The small amounts of flight detected at night (~ 10% during incubation, < 2% during chick-rearing) and foraging (< 1%) could have occurred during twilight. There was no difference in night-time behaviours between species or stages.

All dives were shallow (< 2 m) and there was no difference in diving depth between the two species. During incubation, we found no difference in the number of take-offs and landings on/from the water per day at sea (we had only four chick-rearing trips with immersion data so we excluded these). During daylight, red-tailed tropicbirds had fewer but longer “wet bouts” (mainly time spent sitting on the water) than white-tailed tropicbirds, indicating that they switched between flight and resting on the water less frequently. The opposite happened at night, with white-tailed tropicbirds slightly less active than red-tailed tropicbirds.

### Diet

Sufficient DNA could be extracted to identify prey in only six white-tailed tropicbird and eight red-tailed tropicbird faecal samples, and in two red-tailed tropicbird regurgitates. In addition, we could identify species visually in four white-tailed and two red-tailed tropicbird regurgitates (full list of species and their frequency in Table S7). This limited sample size does not allow any robust comparison of the species’ diets and can only shed light on some prey items. Samples from red-tailed tropicbirds were dominated by flying fish (77.8%), with dolphinfish making up the rest (33%), while in white-tailed tropicbirds, samples mostly contained flying fish (29%), halfbeaks (29%) and goatfish (21%).

### Breeding success and causes of nest failure

**Fig. 4:**
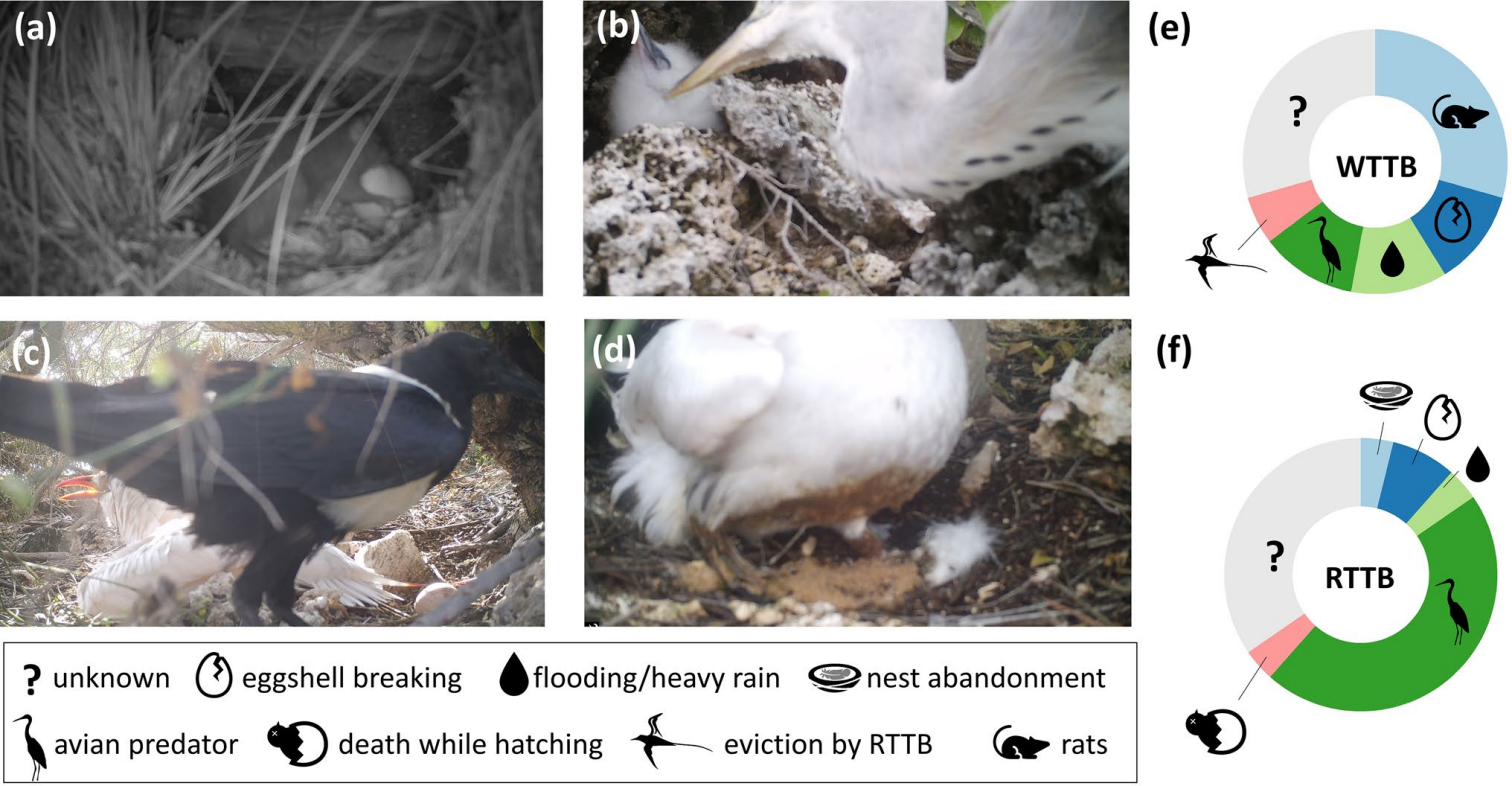
Causes of failure of white-tailed tropicbird and red-tailed tropicbird nests on Aldabra, as recorded by camera traps. In all cases, we chose the most representative photo of the series taken by the camera trap, but the egg or chick were confirmed to have disappeared in subsequent images. **a** Black rat eating a white-tailed tropicbird egg. **b** Grey heron catching a white-tailed tropicbird chick at the nest. **c** Pied crow eating a red-tailed tropicbird egg. **d** Remains of a young red-tailed tropicbird chick after heavy rain. **e**, **f** Causes of failure of white-tailed and red-tailed tropicbird nests, expressed as proportions (WTTB white-tailed tropicbird, RTTB red-tailed tropicbird). Absolute values are in Table 2

The breeding success of white-tailed tropicbirds nests recorded during the study period was very low (10.5%, n = 19; see also Table 2), with red-tailed tropicbird breeding success even worse (3.7%, n = 27; see also Table 2). Predation was a key driver of nest failure for both species (Table 2, Fig. 4). The main predator of white-tailed tropicbirds was black rats, which targeted unattended eggs and were responsible for at least 41% of nest failures. In contrast, 65% of nest failures in red-tailed tropicbirds were due to predation from other native birds, such as grey herons (*Ardea cinerea)* and pied crows (*Corvus corvus)*. These predators attacked eggs and young chicks even in the presence of an adult (our cameras recorded adults under attack from avian predators on 10 occasions (25% of monitored nests), two with an incubating bird and eight with a bird brooding a young chick). Rats were only seen to target unattended eggs, and as red-tailed tropicbirds in our study nests never left their egg alone, they seemed unaffected by them, even on rat-infested islets. We did not see rats take red-tailed tropicbird chicks, despite several chicks being left unattended on islets with confirmed rat presence from the camera traps. We did not see rats take white-tailed tropicbird chicks either, but this may be because all but one white-tailed tropicbird nests which successfully hatched a chick were on islets where rats were not recorded on camera, suggesting that on rat-infested islets, most white-tailed tropicbird nests are already depredated during incubation.

**Table 2:** Breeding success and causes of tropicbird nest failures recorded by camera traps on Aldabra

	White-tailed tropicbird	Red-tailed tropicbird
Breeding success (year-round monitoring 2018–2019)	4.6% (n = 87)	14.5% (n = 69)
Breeding success (our study)	10.5% (n = 19)	3.7% (n = 27)
Hatching success	26.3%	70.4%
Nests with known cause of failure	n = 12	n = 14
Rats eating unattended egg	41% (suspected 50%)	–
Eggshell breaking during manipulation by bird	17%	14%
Weather	17% (high tide)	7% (heavy rain)
Avian predator: total	17%	65%
Grey heron *Ardea cinerea* (on chick)	17%	14% (suspected 43%)
Pied crow *Corvus corvus* (on chick or egg)	–	14%
Malagasy sacred ibis *Threskiornis bernieri* (on chick)	–	7%
Eviction by red-tailed tropicbird	8%	–
Nest abandonment	–	7%
Chick dying while hatching	–	7%

The “suspected 50%” value includes an additional two white-tailed tropicbird nests for which we did not directly see rats take the egg, but we saw the bird leave the egg unattended in the nest, then rats going inside the nest. The “suspected 43%” value includes an additional four red-tailed tropicbird nests for which we did not directly see a heron predate the nest, but we saw the adult tropicbird displaying aggressive behaviour towards a predator coming from above (see examples in Figure S2), during which the chick disappeared. This behaviour was similar to that of tropicbirds under known attacks from herons. For some nests, we could determine nest failure (egg or chick disappeared) but not the cause, most often because the camera did not trigger when the event took place

## Discussion

Our study reveals the foraging movements during breeding of sympatric red-tailed and white-tailed tropicbirds in the Western Indian Ocean. Both species have an extensive foraging range, with red-tailed tropicbirds ranging substantially further, but surprisingly, they are completely segregated in their foraging distribution, despite sharing similar at-sea behaviour.

The complete segregation in foraging areas between the two species is a key finding of our study. Studies elsewhere in the Indian Ocean have shown that these species’ diets largely overlap (Le Corre et al. 2003; Catry et al. 2009), and our diet data, although limited, suggests a similar pattern on Aldabra, with flying fish the dominant prey in both species. Although differences in prey size could contribute to resource partitioning (red-tailed tropicbirds are larger and so may take bigger prey), we were not able to measure this due to the advanced stage of digestion of most diet samples. Such marked spatial segregation between closely-related seabirds from the same colony is rare. Overlap in foraging distribution is common among sympatric seabirds, even when species share a similar diet (Dehnhard et al. 2020; Reisinger et al. 2020). This is also the case in non-avian marine predators (Kiszka et al. 2011; Hoskins et al. 2017). However, most of these studies were conducted in temperate or polar regions.

Studies of tropical species are less common, but two sympatric frigatebird species in the Timor Sea showed high spatial, but low trophic, overlap (Mott et al. 2017), while neighbouring colonies of two booby species with a similar diet in the Caribbean Sea showed marked spatial segregation (Austin et al. 2021), and sympatric booby species in the Western Indian Ocean showed relatively high spatial segregation and diet differences. Studies using stable isotopes to compare diet found segregated trophic niches between various tropical seabird species (Catry et al. 2009; Young et al. 2010; Mancini and Bugoni 2014). The complete segregation observed in our study is likely a mechanism of competition-avoidance, given the similar at-sea behaviour and diet of the two species. The reason behind this apparent tendency for stricter resource partitioning between sympatric species in tropical waters, perhaps linked with lower resource availability in oligotrophic tropical waters, remains unclear. Interestingly, substantial spatial and diet segregation was also found in sympatric subspecies of chick-rearing *Calonectris* shearwaters in the Mediterranean Sea, which is not tropical but is an oligotrophic environment (Navarro et al. 2009). In any case, this resource partitioning may have important implications for tropical species’ conservation, for example by affecting species’ exposure to different threats, or the size of protected areas needed to encompass multiple species’ foraging grounds.

The spatial segregation we observed could also be due to body size differences. The larger size of red-tailed tropicbirds enables them to catch larger prey (Diamond 1975), but may also allow them to reach better foraging areas. This is supported by the fact that red-tailed tropicbirds flew faster and travelled further than white-tailed tropicbirds during both breeding stages, and reached deeper, slightly warmer, and more productive waters during incubation (and to a lesser extent during chick-rearing). Such waters likely host more flying fish (Lewallen et al. 2018), which are key prey for tropicbirds. However, spatial segregation occurred even within distances reachable by white-tailed tropicbirds, suggesting that body size differences alone cannot drive this segregation, and competition avoidance is likely the main mechanism driving interspecific divergence in this system.

The maximum distance travelled by red-tailed tropicbirds from Aldabra is comparable to that of similar-sized red-billed tropicbirds *P. aethereus* tracked in the eastern Atlantic Ocean (Diop et al. 2018) and the Caribbean (Madden et al. 2023). Chick-rearing white-tailed tropicbirds travelled on average 15 km from Aldabra, similar to the shorter trips recorded in the species near Brazil (Campos et al. 2018), but unlike that study, we did not record any multi-day trips. However, due to high nest predation (discussed below), our sample of chick-rearing white-tailed tropicbirds was low and biased towards birds with young chicks, so our data may not be representative of the full chick-rearing period. For both species, incubation shift durations were comparable to those previously recorded on Aldabra (Diamond 1975), while chick-rearing trip durations were similar to those recorded elsewhere in the Indian Ocean (Ramos and Pacheco 2003; Sommerfeld and Hennicke 2010). Unsurprisingly for plunge-diving species, we found that both species were shallow divers and spent the majority of their time at sea in flight during daylight, especially during chick-rearing, reflecting previous findings in red-tailed tropicbirds (Sommerfeld and Hennicke 2010).

Our findings have important conservation implications. First, the large foraging range of both species means that the Aldabra Special Marine Reserve, a no-take zone around Aldabra, only protects a small fraction of the birds’ foraging grounds. The new Spatial Marine Planning Area Zone 1, implemented in 2021, covers a larger proportion, but excludes a large part of the core feeding areas of both species. A substantial proportion of the foraging range of both species (28% and 35% for white-and red-tailed tropicbirds, respectively) was even beyond the Seychelles Exclusive Economic Zone (EEZ). This highlights the need for international cooperation in marine predator conservation, as the waters used by red-tailed tropicbirds northwest of the EEZ are international waters, and those used by white-tailed tropicbirds south of the EEZ are under the jurisdictions of the Comoros, Madagascar, and France. There is little evidence that tropicbirds are at high risk of bycatch by fisheries (only two records to date, Pott and Wiedenfeld 2017) but birds could still suffer indirectly from fisheries. For example, dolphinfishes and rainbow runners, known prey of tropicbirds (Le Corre et al. 2003; Catry et al. 2009) and present in our diet samples, are common bycatch in tuna fisheries (Amandè et al. 2008; Romanov 2008). Furthermore, tropical seabirds often rely on large sub-surface predators like tuna and dolphins to locate and access prey (Miller et al. 2018), so the declining tuna stocks in the Indian Ocean caused by persistent overfishing (Nisar et al. 2021) may be reducing their foraging opportunies.

The spatial segregation between the two tropicbird species also highlights the need for species-specific research to inform local conservation. While our study revealed important foraging grounds for tropicbirds, many other seabird species breed on Aldabra. In particular, the atoll is an important breeding site for great and lesser frigatebirds and red-footed boobies, while greater-crested terns, Caspian terns, black-naped terns, white terns, brown noddies and tropical shearwaters are all frequent breeders (Diamond 1971). With the exception of great frigatebirds (Weimerskirch et al. 2010) and tropicbirds (our study), the foraging movements of most species remain unknown, but several almost certainly forage beyond the Marine Reserve boundaries. This is likely the case for the other pelagic feeders (boobies, shearwaters, white terns, noddies), but perhaps not for the other terns, which usually feed coastally. Investigating the foraging movements of other seabird species during breeding would provide a clearer picture of the extent of the foraging grounds of the Aldabra seabird community and of the threats birds may face at sea. Additionally, because our study focuses on a single season (the northwest monsoon) in a single year, research investigating inter-annual and inter-seasonal variation in tropicbird foraging distribution would shed more light on the species’ foraging area use in the region.

The differences in foraging range and spatial segregation between the two species are, however, unlikely to explain their contrasting trends in the number of breeding attempts on Aldabra (stable in white-tailed tropicbirds, declining in red-tailed tropicbirds, SIF monitoring, unpubl. data). While red-tailed tropicbirds foraged further offshore, which can sometimes indicate food shortages near the colony and lead to low breeding success and ultimately population declines (Fayet et al. 2021), we do not believe that food shortages are the cause of the observed population decline on Aldabra. Our camera traps recorded many instances of parents feeding their chicks, which seemed to grow well, and we did not observe starving chicks during the study. Instead, predation was the main driver of low breeding success in red-tailed tropicbirds (65% of nest failures caused by predation, recorded on camera). Its main cause was native avian predators, particularly grey herons, and to a lesser extent pied crows, both of which prey on other birds on Aldabra (Wanless and Jupiter 2002; Pistorius 2008). This is particularly interesting, because unlike many other red-tailed tropicbird colonies, Aldabra still has its original native predator community, which allows us to quantify predation in a relatively undisturbed environment. Predation of red-tailed tropicbird chicks usually occurred when chicks were young and often in the presence of an adult bird. All 10 recorded attacks of red-tailed tropicbird nests by an avian predator showed the adult tropicbirds being aggressive towards the predator but ultimately unable to defend their nest (on one occasion, crows were deterred but returned later, this time successfully). This is perhaps not surprising, given the substantially larger size of the predators and the potential injuries they could inflict to the adult bird. Other relatively large seabirds are also generally unable to defend their nest against large avian predators (e.g. Descamps et al. 2005; Veitch et al. 2016).

While we found no obvious link between the tropicbirds’ foraging trips and their breeding success, another type of movement, only seen in white-tailed tropicbirds, appeared related to nest failure. During incubation, some white-tailed tropicbirds took very short trips, leaving their egg unattended, which made them highly susceptible to black rat predation. The function of these trips, whether foraging or perhaps cooling or cleaning, remains unclear. Egg neglect is widespread in seabirds, including tropicbirds (Saunier et al. 2022), and the eggs of many species—especially those with long incubation stints like Procellariforms—have even evolved a tolerance to temporary chilling (Boersma and Wheelwright 1979). However, this behaviour becomes risky in the presence of invasive terrestrial predators such as rats or mice (Blight et al. 1999; Saunier et al. 2022). This was the case on Aldabra, where the main driver of low breeding success in white-tailed tropicbirds was egg predation by rats, which took place only when the egg was unattended (despite many instances of rats seen close to incubating adults). Red-tailed tropicbirds on Aldabra did not leave their egg unattended, although they do elsewhere (Saunier et al. 2022), which would explain why their nests were not targeted by rats even on rat-infested islets.

In contrast to red-tailed tropicbirds, white-tailed tropicbird nests were less susceptible to avian predators. This likely stems from differences in nest site preferences, with the more open nests of red-tailed tropicbirds likely more visible and vulnerable to avian predators than the crevices in which white-tailed tropicbirds prefer to breed. This is supported by an experimental study in Chile, which found evidence of avian predators only visiting more exposed red-tailed tropicbird nests (Luna et al. 2018). Such high predation pressure, added to other failures caused by bad weather, egg breakage or abandonment, led to substantially lower breeding success than previously recorded in both species (Prys-Jones and Peet 1980; Burt et al. 2021). This could explain the decline in red-tailed tropicbird breeding activity observed on Aldabra since the 2010s, through lack of recruitment. The ability of white-tailed tropicbirds to breed year-round, and their shorter breeding cycle, may make their population more resilient to such low breeding success, but we can expect further declines and local extinctions from some parts of the island in future, if rat predation continues unchecked.

The scale of nest predation revealed by our study highlights the substantial benefits that measures to reduce predation would have on Aldabra’s tropicbird populations. For instance, control of invasive terrestrial predators on a red-tailed tropicbird colony in Hawai’i led to a yearly population growth of 11% (Vanderwerf 2021). Rats are invasive on Aldabra and impact many other species, including birds, giant tortoises and plants (Harper and Bunbury 2015), and their eradication would have a huge impact on the entire island ecosystem. Eradicating rats from tropical islands is notoriously difficult (Keitt et al. 2015), but techniques are improving and several successful eradications have been achieved (Griffiths et al. 2019). Measures to protect red-tailed tropicbird nests from native avian predators are also sorely needed, especially as Aldabra is a major breeding site for the species in the region (Diamond 1971; Schreiber and Schreiber 2020). It is possible that eradicating rats would also help reduce avian predation, by increasing suitable nesting habitat on the main islands of the atoll. Otherwise, non-lethal methods could be trialled, such as creating predator-proof nests, which have been used successfully on cavity-nesting seabirds (Bolton et al. 2004) but not yet, to our knowledge, on ground-nesting species. Deterrents such as decoys may also be an alternative, although their long-term efficiency in unknown.

Our findings provide new insights into the foraging ecology of tropicbirds breeding in the Western Indian Ocean, and into the evolved response to competition by sympatric, closely-related marine predators. By highlighting their extensive feeding ranges during breeding and their sensitivity to different predators, our study also provides critical information for the conservation of tropicbirds on Aldabra and elsewhere and sheds new light on the potentially disastrous effects of predation by a range of introduced and native predators on tropical seabirds. With seabirds declining globally at an alarming rate, measures to make important seabird colonies like Aldabra safe havens for breeding seabirds are more urgent than ever.

### Supplementary Information

Below is the link to the electronic supplementary material.

Supplementary file1 (PDF 654 KB)

## Data Availability

Data are available from the BirdLife Seabird Tracking Database (https://www.seabirdtracking.org/) and the Dryad Digital Repository (https://doi.org/10.5061/dryad.fttdz0909, Fayet et al. 2023).
