# Supplementary material for: Marked differences in foraging area use and susceptibility to predation between two closely-related tropical seabirds
Source: Oecologia. 2023 Oct 10;203(1-2):167–79. doi: 10.1007/s00442-023-05459-x (PMC10615964; doi:10.1007/s00442-023-05459-x)
Supplement: Supplementary file 1 — Supplementary file1 (PDF 654 KB) [file 442_2023_5459_MOESM1_ESM.pdf]

Electronic Supplementary Material for:

Fayet AL, Sanchez C, Appoo J, Constance J, Clucas G, Turnbull LA & Bunbury N (2023) Marked differences in foraging area use and susceptibility to predation between two closely-related tropical seabirds. *Oecologia*

Contents

Dietary metabarcoding methods ……………………………………………………………2

Behavioural classification ……………………………………………………………5

Table S1 ……………………………………………………………7

Table S2 ……………………………………………………………7

Figure S0 ……………………………………………………………8

Table S3 ……………………………………………………………12

Table S4 ……………………………………………………………12

Table S5 ……………………………………………………………13

Table S6 ……………………………………………………………14

Table S7 ……………………………………………………………14

Figure S1……………………………………………………………16

Figure S2……………………………………………………………17

Figure S3……………………………………………………………18

References ……………………………………………………………18

Dietary metabarcoding methods

DNA extractions from the faecal samples were performed using Zymo Research (Irving, CA) Quick-DNA Fecal/Soil Miniprep kits. Samples and field blanks were defrosted, vortexed, and centrifuged for 2 min at 8,000 g to collect all material to the bottom of the tubes. The RNA Later preservation buffer was pipetted off, and up to 150 mg of the faecal material was transferred to the kit's bead bashing tubes. Where samples were very small, the RNA Later was removed from the tube and the tube was washed out with the bead bashing buffer, to transfer as much faecal material as possible to the bead bashing tube. This was also the method used for the field blanks, where no faecal material was present. Bead beating took place on a benchtop vortex set at maximum speed for 20 minutes. The rest of the DNA extraction was performed following the manufacturer's instructions, eluting the DNA in a final volume of 60 ul of elution buffer. Extractions were performed in three batches, with samples randomly mixed among batches to avoid batch effects, and a negative control (extraction blank) included with each. All DNA extractions and PCR set-ups were performed in a dedicated clean lab.

Metabarcoding was performed with a 2-step PCR approach. In the first step, the 12S rRNA gene was amplified using MiFish primers (Miya et al., 2015) with overhanging Truseq tails. The forward primer sequence was: 5'-ACACTCTTTCCCTACACGACGCTCTTCCGATCTGTCGGTAAAACTCGTGCCAGC-3'; and the reverse primer sequence was: 5'-GTGACTGGAGTTCAGACGTGTGCTCTTCCGATCTCATAGTGGGGTATCTAATCCCAGTTTG-3'. We included a PCR-blank with every batch of PCRs to monitor for contamination during set-up. A mock community (positive control), including DNA extracted directly from fish samples, was set-up last, in our main, pre-PCR lab and added to the plate or column after all other PCR tubes had been sealed. The PCR reaction included 6 µL of KAPA HiFi HotStart ReadyMix 2X (KAPA Biosystems, Wilmington, Massachusetts), 0.7 µL of each of the forward and reverse primers at 5 µM concentration, and 4.6 µL of faecal DNA template (or molecular grade water for the PCR-blank). For the mock community, we

reduced the amount of template DNA to 1 µL and added 3.6 µL of water. Thermocycling conditions were: 95 °C for 3 mins, 35 cycles of 98 °C for 30 secs, 65 °C for 30 secs, 72 °C for 30 secs, followed by a 5 minute extension at 72 °C. PCR products were visualised using electrophoresis in a post-PCR lab to ensure that all blanks were clear. PCRs were performed in triplicate, with the products from three replicates being combined and diluted 1:10 before the second stage PCR.

The second stage PCR used the diluted product from the first stage as template and added the flow cell binding sites and sequencing primer binding sites. The primer sequences were: forward, 5'-AATGATACGGCGACCACCGAGATCTACAXXXXXXXXACACTCTTTCCCTACACGAC-3' and reverse, 5'-CAAGCAGAAGACGGCATACGAGATXXXXXXXXGTGACTGGAGTTCAGACGTGT-3'. The octo-X sites represent the i7 and i5 indexes used to identify samples. We used unique dual indexes, such that any reads that suffered from tag-jumping would be eliminated during de-multiplexing. The PCR reaction used 6 µL of KAPA HiFi HotStart ReadyMix 2X, 0.7 µL of each of the forward and reverse primers at 5 µM concentration, 1 µL of template and 3.6 µl of molecular grade water. The thermal cycling profile was 94 °C for 3 mins, 12 cycles of 94 °C for 30 secs, 50 °C for 30 secs, 72 °C for 30 secs, and a final extension of 7 mins at 72 °C.

Sequencing was performed on an Illumina HiSeq 2500 using Rapid Run chemistry with 250bp paired-end reads at the University of New Hampshire's Hubbard Center for Genome Studies. Samples were loaded alongside other projects, such that each sample was sequenced on approximately 0.02% of a lane. Demultiplexed reads were imported into Qiime2 v2019.4 (Bolyen et al., 2019) and primers at the 3' end were trimmed using the cutadapt plugin (Martin, 2011). The DADA2 plugin (Callahan et al., 2016) was used to denoise and merge reads, running it separately for each lane of samples. During this step, we also trimmed the 5' ends of reads to remove primers and truncated forward and reverse reads to 134 bp and 132 bp, respectively. These truncation lengths were found to maximise the number of sequences that were retained after quality filtering while providing sufficient length for merging. We also truncated reads when the base quality score fell below two. If this truncation resulted in a read shorter than the truncation parameters above, then the read was discarded. All

other settings were left as defaults. After denoising, we merged the samples from different sequencing lanes using the feature-table plugin.

Taxonomy was assigned using a custom script and database. The database included all vertebrate 12S and mitochondrial sequences, excluding primates, downloaded from GenBank on 15/12/2017. Taxonomy assignments were made using an iterative blast method; each representative sequence was blasted 80 times against the reference database, increasing the percent identity incrementally from 70 to 100% over the 80 iterations. The hit with the highest percent identity was kept. This method circumvents a limitation of the blast method, which keeps only the first hit that meets the search criteria, rather than the best hit. The minimum query coverage was set to 40%. This script is available from https://bitbucket.org/dwthomas/qiime2_tools/src/master/mktaxa.py.

Avian, mammalian, and unassigned sequences were filtered out using the taxa plugin and alpha diversity rarefaction curves were calculated using the diversity plugin. We found that the number of observed taxa levelled off above a sequencing depth of approximately 1500 reads for faecal samples, and at approximately 3000 for the mock community. This discrepancy was because our mock community was artificially super-diverse relative to the faecal samples. Nevertheless, we decided to re-sequence samples with fewer than 3000 prey reads to ensure that we had captured the entire diversity of the samples. We repeated all the above steps to combine the re-sequenced reads with the original reads from repeated samples.

After resequencing, the diversity captured in faecal samples still plateaued at approximately 1500 reads and so we rarefied our final feature table to a depth of 1500 reads using the feature-table plugin. Notably, there were no fish sequences picked up by any of our field-, extraction-, or PCR-blanks, (a few avian and mammalian sequences were picked up, but these did not make it past our filtering) and so we did not make any adjustments for contamination when analysing our results.

We then manually checked all taxonomy assignments using NCBI’s blastn suite (https://blast.ncbi.nlm.nih.gov) in December 2020 and checked the geographic distributions of each

fish species using FishBase (https://www.fishbase.de). If a percent identity was less than 97%, then a genus- or family-level assignment was made depending on the alternate hits and percent identity. If there was more than one species assignment greater than 97% and those taxa occurred in the Indian Ocean, then we made a genus or family-level assignment based on the hits. Alternatively, if only one of the possible species was found in the Indian Ocean, then we made a species-level assignment.

Behavioural classification

<u>Overview</u>

The accelerometer and dive data (n=10 birds) were classified in behavioural classes using an unsupervised machine learning approach; the results were then used to label the GPS and light/immersion data collected from the same birds. The labelled data was then used to train a supervised machine learning model to classify behaviour from the rest of the data from the (n=20) birds tracked with a GPS and or GPS + geolocator.

The accelerometer and dive data were used to calculate 65 variables of potential relevance to classifying behaviour (Table S1), based on their use for this aim in other studies (Nathan et al., 2012; Patterson et al., 2019; Shamoun-Baranes et al., 2012). These variables were then averaged over 1 second. 26 were subsequently dropped because of high correlation ($|r| > 0.9$) with one or more of the retained variables, leaving 39 variables (Table S1). The light and immersion data from the geolocators were used to calculate 18 variables over 1-min windows, 11 were subsequently dropped because of high correlation ($|r| > 0.9$) with others, leaving seven variables (Table S2). The GPS data were interpolated to 1-min intervals using continuous-time correlated random walks from the *crawl* package. Speed, turning angle, tortuosity, distance from the nest, distance covered since the previous location, and distance covered since start of the trip, were then calculated for each location.

The 39 accelerometer data variables, along with the seven light and immersion variables, were scaled then inputted in a k-means clustering algorithm along with binary variables 'species' and 'day/night' (estimated from time of day) and the continuous variable 'hours since sunset'. We tested

between two and 20 clusters, retaining six as the optimal number based on the total within-sum-of-squares, the gap statistic, and silhouette scores. Based on the characteristics of the variables in these clusters as well as their geographical location (see details below), we concluded that they represented the following behaviours: ‘at the nest’, ‘foraging’, ‘sitting on the sea surface during the day’, ‘sitting on the sea surface at night’, and two types of flights which mainly differed in wingbeat frequency but which we grouped together in the subsequent analysis as ‘flight’ for simplicity.

These datapoints were then labelled accordingly and used to train a supervised model, this time using only GPS and geolocator variables (see details below about model training and testing). The data were split into an 80:20 training and testing set. We compared the performance of Naïve Bayes, Multivariate Adaptive Regression Splines (MARS), Neural Network and Gradient Boosted Trees models. For each model, the hyperparameters were tuned using ten-fold cross validation and the best model selected using the roc_auc metric (all variables were scaled and centered within the cross-validation process). When inspecting accuracy of the tuned models on the held-out test set, all models performed well (accuracy > 88%). The gradient boosted tree model had the highest performance (accuracy = 94.5%, macro F1 = 92.2%) and was retained (details below). This model was then used to predict behaviour in the data from the birds fitted with a GPS and geolocator. A second gradient boosted tree model was trained on the GPS-only variables (accuracy = 88.9%, macro F1 = 85.7%) and used to predict behaviour in the data from the birds fitted with a GPS device only.

Table S1. Accelerometer variables retained and dropped from the analysis. * indicates variables calculated over a 2-second moving window, # indicates variables calculated over a 5-second moving window. (X, Y and/or Z) indicates the variable was calculated along each axis, q indicates the variable was calculated along the length of the diagonal of the x-y-z volume.

**Retained variables**	
Azimuth	Pitch
Azimuth circular variance*	Pressure (from depth logger)
Depth (from depth logger)	q (square root of the sum of squares of X, Y, Z)
Dynamic acceleration* (X, Y, Z)	Skewness* (X, Y, Z, q)
Inclination angle	Static acceleration* (Y, Z)
Kurtosis* (X, Y, Z)	Surge frequency#
Maximum dynamic acceleration* (X)	Sway frequency#
Maximum acceleration* (X, Y)	Temperature (from temperature logger)
Mean dynamic acceleration* (X, Y, Z)	Trend in raw acceleration# (X, Y, Z, q)
Minimum acceleration* (X, Y, q)	Vertical speed (change in depth / sec)
Number of dives (from depth logger)	Wing beat frequency#
Overall dynamic body acceleration (ODBA)	Yaw
**Dropped variables**	
Inclination circular variance*	Standard deviation of ODBA*
Kurtosis (q)	Standard deviation of raw acceleration* (X, Y, Z, q)
Maximum dynamic acceleration* (Y, Z)	Smoothed pitch*
Maximum acceleration* (q, Z)	Smoothed roll*
Minimum dynamic acceleration* (X, Y, Z)	Smoothed yaw*
Minimum acceleration* (Z)	Static acceleration* (X)
Roll	Raw acceleration (X, Y, Z)
Standard deviation of dynamic acceleration* (X, Y, Z)	

Table S2. Geolocator variables retained and dropped from the analysis.

**Retained variables**	
Total time wet	Total number of bouts
Number of landings (dry -> wet)	Mean light level
Number of take-offs (wet -> dry)	Sd of light level
Number of dry bouts (≥ 6s dry)	
**Dropped variables**	
Total time dry	Minimum dry bout duration
Proportion of time wet	Maximum dry bout duration
Number of wet bouts (≥ 6s wet)	Mean dry bout duration
Minimum wet bout duration	Minimum light level
Maximum wet bout duration	Maximum light level
Mean wet bout duration	

Step 1 – identifying behaviour in the accelerometer & immersion data

We implemented a k-means clustering algorithm to classify the data from the 10 birds fitted with accelerometers (8 of which also had an immersion logger). For this we used the 39 accelerometer data variables and the 7 GLS variables described above. All continuous variables were scaled. Two binary variables ‘species’ and ‘day/night’ (estimated from time of day) and the continuous variable ‘hours since sunset’ were also added.

To determine the optimal number of clusters, we tested between two and 20 clusters. The location of the knee point on the total within sum-of-squares (Fig S1.A) suggested an optimal number of clusters of five or six. We decided for six based on the Hubert statistic (Fig S1.B). Additionally support for six clusters came from the silhouette score which was slightly higher for six clusters (0.149) than for five (0.138).

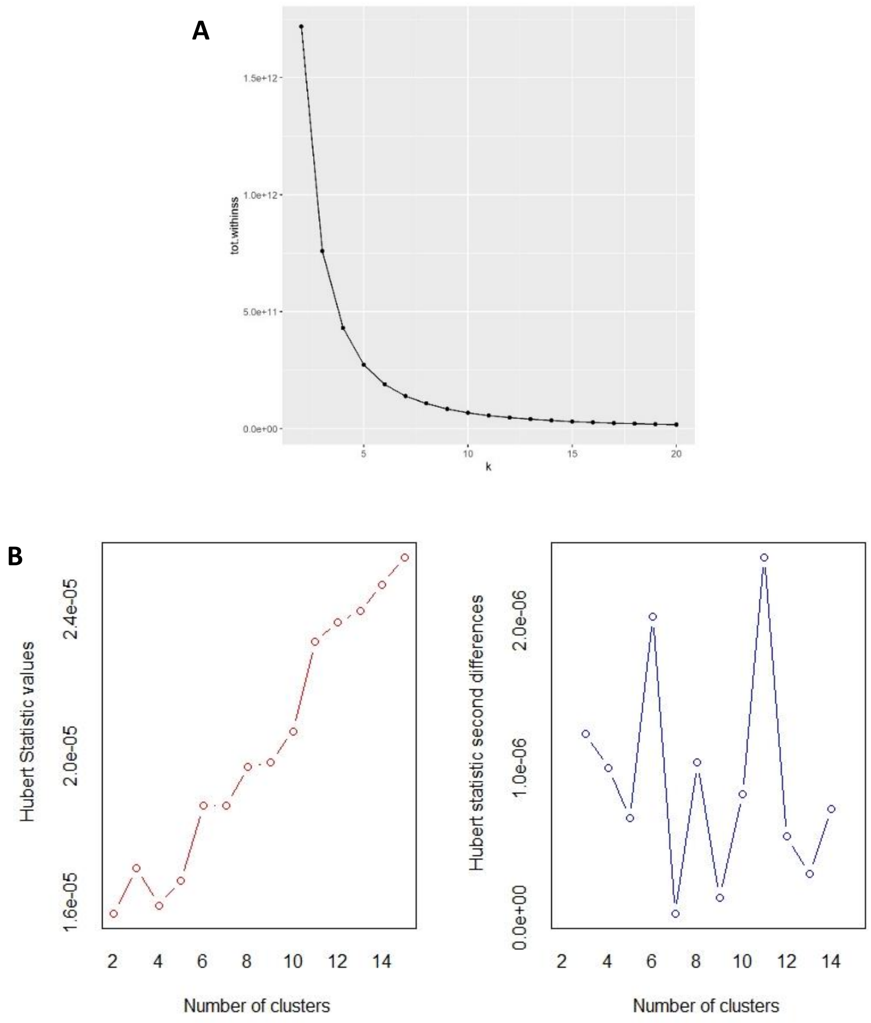

Figure S0. (A) Total within sum-of-squares from the kmeans clustering for 2-20 clusters. The location of the knee point suggests an optimal number of 5 or 6 clusters. (B) Hubert statistic for 2-15 clusters (left) and second difference (right). The sharp increase in the statistic between 5 and 6 suggests 6 clusters is better than 5.

We then explored the characteristics of the variables in each cluster to identify which behaviour each most likely represented. We identified the behaviours as follow (values are means ± SE).

Cluster 1 (16.6 % of data points) took place very close to the nest (0.2 ± 0.005 km). Based on the very low speed (0.1 ± 0.04 km/h), lack of wing flapping (WBF = 0.001 ± 0.0002 Hz) and very low ODBA (0.03 ± 0.0003 g), we concluded the birds were stationary. The birds were also always dry (total time wet = 0.02 ± 0.009 sec / min) and no diving occurred (not a single dive recorded in 9,785 minutes). We concluded this behaviour corresponded to the birds being **at their nest**.

Cluster 2 (2.9 % of data points) took place at sea (on average > 200km away from the nest), at medium speed (10.6 ± 2.3 km/h) and with a relatively high wing beat frequency = 2.2 ± 0.05 Hz and a high ODBA (0.8 ± 0.01 g). Furthermore, the immersion data showed the birds were getting in and out of the water during this behaviour (total time wet = 30.0 ± 0.4 sec / min, 0.8 ± 0.01 landing /min, 0.7 ± 0.01 take-off /min, number of bouts = 2.5 ± 0.02 / min), which is the only cluster in which this happened. The highest dive number occurred during this cluster (101 dives recorded in 1,726 minutes). We concluded this behaviour was related to **foraging**. Due to its low occurrence, we believe this is a specific aspect of foraging, possibly when birds are pursuing a prey and then entering the water to catch it, i.e. diving from the air. Based on current knowledge of tropicbird foraging behaviour, this would therefore not include foraging when birds catch prey in the air (without landing on the water) or when they dive from the water surface.

Cluster 3 (24.0 % of data points) occurred mostly during the day (98.9 %) and took place at sea (on average > 200km away from the nest), at very low speed (2.1 ± 0.03 km/h) and without wing

flapping (WBF = 0.04 ± 0.001 Hz) and with a low ODBA (0.27 ± 0.001 g). In addition, the birds were always wet in this behaviour (total time wet = 59.4 ± 0.05 sec / min) and there were no take-offs or landings (<0.01 landings or take-offs / min) nor diving occurs (11 dives recorded in 14,159 minutes). These characteristics are consistent with the **birds sitting on the water during the daytime**, where the bobbing movement on the water and the currents explain the small amount of movement.

Cluster 4 (34.8 % of data points) is similar to Cluster 3 but occurred mostly at night (91.3 %). Like cluster 3, it took place at sea (on average > 200km away from the nest), at very low speed (1.3 ± 0.01 km/h), without any wing flapping (WBF = 0.001 ± 0.0008 Hz), and with low ODBA (0.2 ± 0.0004 g), and the birds were always wet (total time wet = 59.7 ± 0.03 sec / min) and did not take-off or land (<0.01 landings or take-offs / min). Diving was rare (115 dives recorded in 20,508 minutes). We concluded this behaviour represented the birds **sitting on the water at night.**

Cluster 5 (14.8 % of data points) took place at sea (on average > 200km away from the nest), with relatively highly speed (19.1 ± 1.2 km/h) and high wing beat frequency (4.0 ± 0.01 Hz) and ODBA (1.0 ± 0.003 g). The birds were almost always dry (total time wet = 3.6 ± 0.2 sec / min) and almost never took off or landed on the water (<0.01 landings or take-offs / min). However some diving occurred (177 dives recorded in 8,747 minutes). We concluded this behaviour corresponded to the birds being in **flapping flight.**

Cluster 6 (6.7 % of data points) also took place at sea (on average > 200km away from the nest), with relatively highly speed (26.0 ± 8.9 km/h) but, unlike the flapping flight above, with low wing beat frequency (0.3 ± 0.01 Hz) and lower ODBA (0.25 ± 0.002 g). Birds were always dry (total time wet = 0.2 ± 0.05 sec / min) and there was no take-offs or landings (<0.01 landings or take-offs / min) nor any diving (not a single dive recorded in 4,045 minutes). Based on this, we suggest this corresponds to a rarer type of flight which is **gliding flight**.

<u>Step 2 – training a machine learning model to identify this behaviour in GPS & GLS data</u>

All these datapoints were then labelled according to their behaviour and were used to train a supervised model using the R package *tidymodels*. For this model, we only included variables from GPS and GLS variables (the 20 GLS variables described earlier and the six GPS variables described in the main text). Species, day/night and hours since sunset were also included. The data were split into a 20-80 training and testing set. We compared the performance of four models: Naïve Bayes, Multivariate Adaptive Regression Splines (MARS), Neural Network and Gradient Boosted Trees models.

For each model, the hyperparameters were tuned using ten-fold cross validation and the best model selected using the roc_auc metric (all variables were scaled and centred within the cross validation process). The characteristics of all models are in Table S3. All models performed well but the model with the highest accuracy, the gradient boosted tree model was retained. The tuned gradient boosted tree model was then used to predict the behaviour of each datapoint from the birds fitted with a GPS and GLS.

As a subsample of the birds were tracked with a GPS only, a second gradient boosted tree model was trained on the GPS variables only (as well as Species, day/night and hours since sunset). This model and used to predict behaviour in the data from the birds fitted with a GPS device only.

The confusion matrices of the final gradient boosted tree models (Tables S4 and S5) show that the main misidentification of behaviour occurs between the two types of flight. Additionally, the confusion matrix for the GPS only model shows that unsurprisingly, the behaviours which are distinguished partly by wet/dry metrics are slightly less well identified. As distinguishing between types of flight was not necessary for our study, we merged the two flight categories together, so that any datapoint assigned either ‘flapping’ or ‘gliding’ flight simply became ‘flight’. This improved the model accuracies to 97.3% for the model trained on GPS + GLS data and to 92.5% for the model trained on GPS data only.

Table S3. Model parameters for the supervised machine learning step

Model	Tuned Hyperparameters	Accuracy (95% CI)	No Information Rate	Macro F1
Naïve Bayes	smoothness = 0.5<br>Laplace = 0	0.795<br>(0.788, 0.802)	0.347	0.673
MARS	degree = 1<br>nprune = 17	0.895<br>(0.889, 0.900)	0.347	0.769
Neural Network	hidden_units = 10<br>penalty = 2.154e-07<br>epochs = 670	0.909<br>(0.904, 0.915)	0.347	0.846
Gradient Boosted Trees	mtry = 11<br>trees = 1000<br>min_n = 7<br>tree_depth = 8<br>learn_rate = 0.022<br>loss_reduction = 2.065e-09<br>sample_size = 0.833	0.945<br>(0.941, 0.949)	0.347	0.922
Gradient Boosted Trees (GPS only)	mtry = 10<br>trees = 1000<br>min_n = 15<br>tree_depth = 8<br>learn_rate = 0.085<br>loss_reduction = 6.098<br>sample_size = 0.583	0.889<br>(0.883, 0.894)	0.347	0.857

Table S4. Confusion Matrix for the final Gradient Boosted Tree model trained on GPS + GLS data. The main misallocation errors related to confusion between the flapping and the gliding flight.

	flap_flight	foraging	glide_flight	nest	seasit_day	seasit_night
flap_flight	1545	0	218	0	22	3
foraging	0	346	4	0	19	4
glide_flight	104	0	600	0	1	0
nest	1	0	0	1949	7	22
seasit_day	78	6	2	0	2685	44
seasit_night	21	2	0	1	85	4019

Table S5. Confusion Matrix for the final Gradient Boosted Tree model trained on GPS data only. The main misallocation errors related to confusion between the flapping and the gliding flight.

	flap_flight	foraging	glide_flight	nest	seasit_day	seasit_night
flap_flight	1396	173	296	0	131	27
foraging	0	0	0	0	1	0
glide_flight	137	20	508	0	11	2
nest	4	1	0	1937	1	11
seasit_day	145	92	25	0	2624	39
seasit_night	69	51	3	2	70	4013

Table S6. Average duration (in hours) engaged in different behaviours per trip for both tropicbird species.

Trip metric	White-tailed tropicbird	Red-tailed tropicbird
Time in flight during daylight (hrs)	25.6 ± 4.5 (INC)<br>2.5 ± 0.9 (CR)	38.5 ± 3.3 (INC)<br>13.8 ± 6.9 (CR)
Time foraging during daylight (hrs)	2.3 ± 0.7 (INC)<br>0.1 ± 0.08 (CR)	2.9 ± 0.5 (INC)<br>0.7 ± 0.6 (CR)
Time sitting on the water during daylight (hrs)	28.7 ± 6.5 (INC)<br>0.6 ± 0.9 (CR)	40.9 ± 3.5 (INC)<br>12.7 ± 7.1 (CR)
Time in flight during night-time (hrs)	1.5 ± 0.8 (INC)<br>0 ± 0 (CR)	3.3 ± 0.6 (INC)<br>0.5 ± 0.5 (CR)
Time foraging during night-time (hrs)	0.4 ± 0.1 (INC)<br>0.01 ± 0.01 (CR)	0.5 ± 0.1 (INC)<br>0.06 ± 0.06 (CR)
Time sitting on the water during night-time (hrs)	46.2 ± 9.7 (INC)<br>3.3 ± 2.1 (CR)	59.8 ± 4.9 (INC)<br>21.9 ± 11.6 (CR)

Table S7. Composition of faecal samples and regurgitates from tropicbirds on Aldabra. Note we only used primers to detect fish, so we did not detect any non-fish species which may have been in the diet, such as squid.

Bird Species	Sample type	Fish species (common name)	Number of samples
Red-tailed tropicbird	Faecal	*Decapterus macarellus* (Mackerel scad)	1
Red-tailed tropicbird	Faecal	*Coryphaena equiselis* (Pompano dolphinfish)	2
Red-tailed tropicbird	Faecal	*Coryphaena hippurus* (Common dolphinfish)	1
Red-tailed tropicbird	Faecal	*Cheilopogon sp.* (Flyingfish sp.)	5
Red-tailed tropicbird	Faecal	*Exocoetus volitans* (Tropical two-wing flyingfish)	1
Red-tailed tropicbird	Faecal	*Hirundichthys sp.* (Flyingfish)	3
Red-tailed tropicbird	Faecal	*Oxyporhamphus micropterus micropterus* (Bigwing halfbeak)	1
Red-tailed tropicbird	Regurgigate	Tetraodontidae sp. (Puffer fish sp.)	1
Red-tailed tropicbird	Regurgigate	Exocoetidae indet. (Flying fish)	1
White-tailed tropicbird	Faecal	*Gymnothorax undulatus* (Undulated moray)	1
White-tailed tropicbird	Faecal	*Heteropriacanthus cruentatus* (Glasseye)	1

White-tailed tropicbird	Faecal	*Exocoetus volitans* (Tropical two-wing flyingfish)	1
White-tailed tropicbird	Faecal	*Exocoetidae indet.* (Flying fish)	1
White-tailed tropicbird	Faecal	*Hemiramphus sp.* (Halfbeak)	1
White-tailed tropicbird	Faecal	*Hemiramphus lutkei* (Lutke's halfbeak)	1
White-tailed tropicbird	Faecal	*Cirripectes sp.* (Combtooth blenny)	1
White-tailed tropicbird	Faecal	*Gempylus serpens* (Snake mackerel)	1
White-tailed tropicbird	Faecal	*Psenes cyanophrys* (Freckled driftfish)	1
White-tailed tropicbird	Faecal	*Oxyporhamphus micropterus micropterus* (Bigwing halfbeak)	2
White-tailed tropicbird	Faecal	*Mulloidichthys flavolineatus* (Yellowstripe goatfish)	1
White-tailed tropicbird	Faecal	*Parupeneus multifasciatus* (Manybar goatfish)	1
White-tailed tropicbird	Faecal	*Parupeneus jansenii* (Jansen's goatfish)	1
White-tailed tropicbird	Regurgigate	*Kuhlia sp.* (Flagtail sp.)	1
White-tailed tropicbird	Regurgigate	*Elagatis bipinnulata* (Rainbow runner)	1
White-tailed tropicbird	Regurgigate	*Exocoetidae indet.* (Flying fish sp.)	2

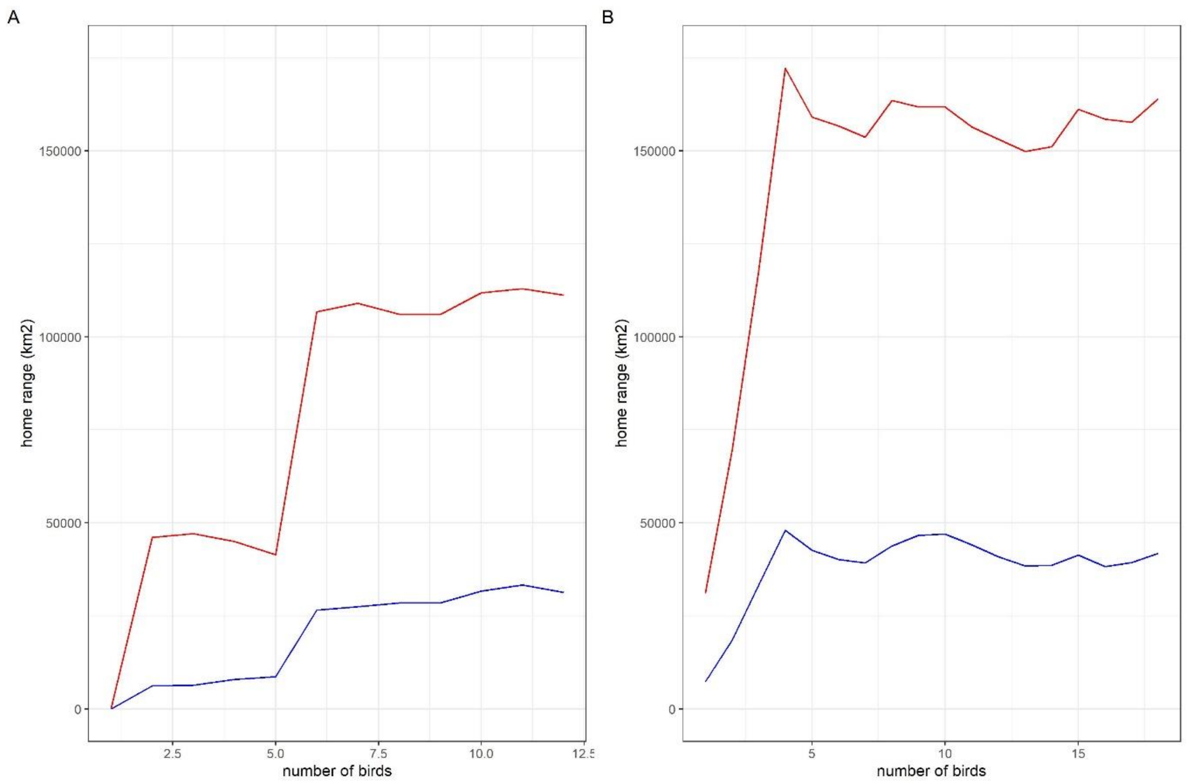

Figure S1. Increase in home range with increasing sample size for (A) white-tailed tropicbirds and (B) red-tailed tropicbirds, for the 50% (blue) and 95% (red) density kernels. For both species, the home ranges plateau well before we reach our maximal sample size, showing that we have obtained a representative home range for the population despite our moderate sample size.

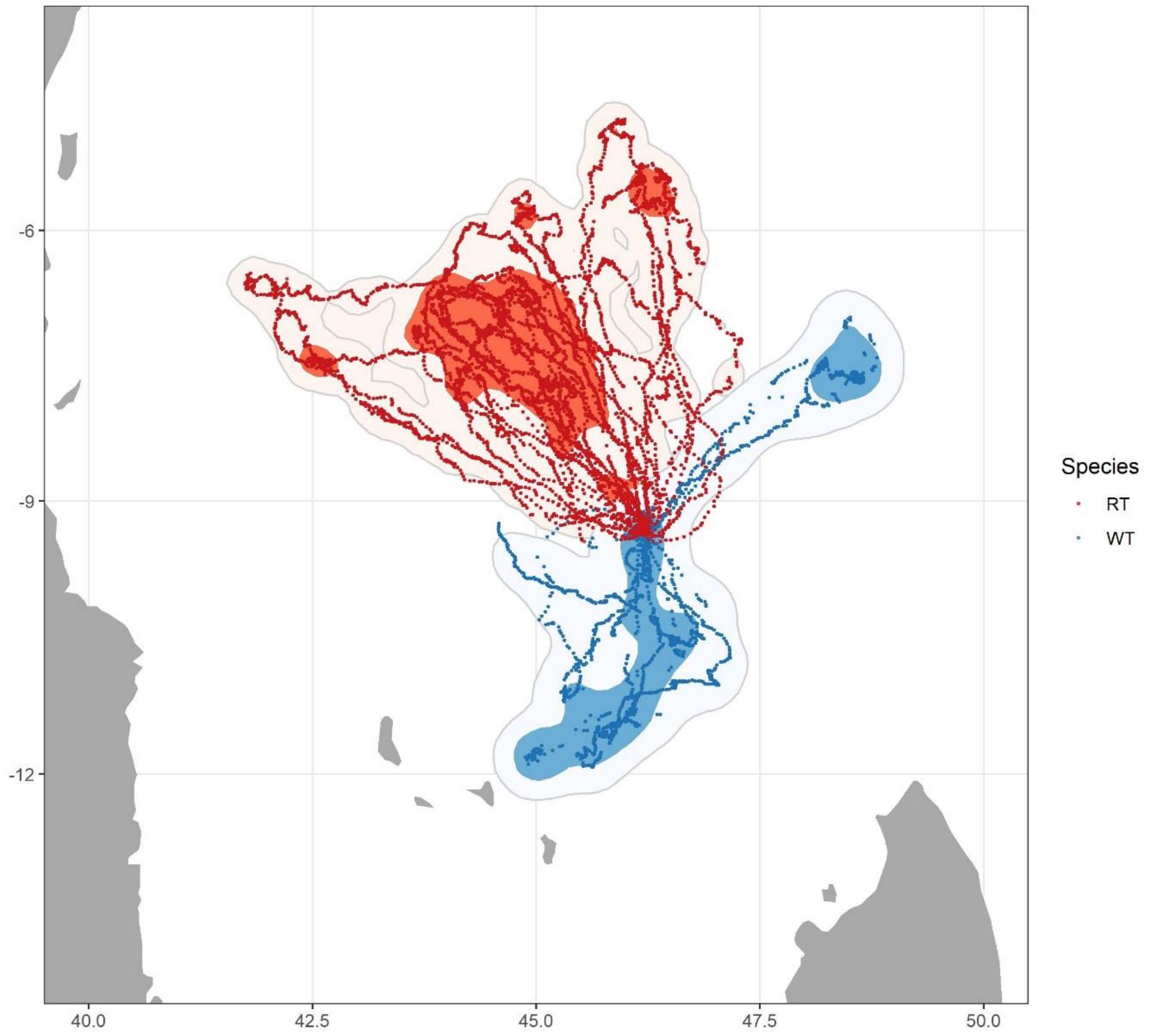

Figure S2. Distribution of red-tailed tropicbirds (red) and white-tailed tropicbirds (blue) during their foraging trips around Aldabra. The dots represent the GPS locations, the pale areas the 95% density kernels, and the darker areas the core foraging areas (50% density contours).

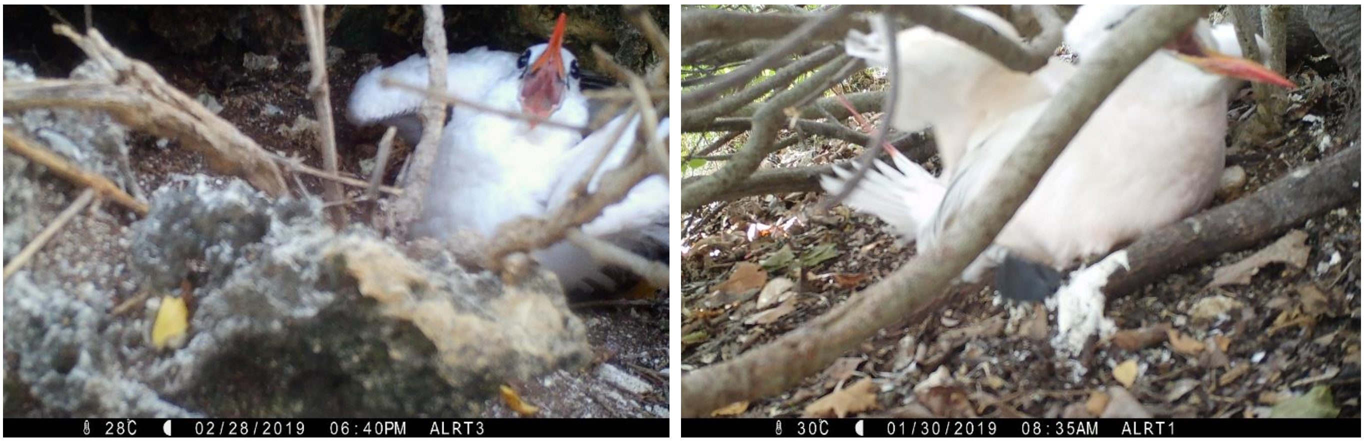

Figure S3. Images of suspected attacks by a heron on red-tailed tropicbird nests. Although the predator is not seen on camera, the tropicbirds are displaying aggression towards something coming from above. In all instances, the chick was not seen again on camera after this, and the adult soon abandoned the nest.

References

Nathan, R., Spiegel, O., Fortmann-Roe, S., Harel, R., Wikelski, M., & Getz, W. M. (2012). Using tri-axial acceleration data to identify behavioral modes of free-ranging animals: General concepts and tools illustrated for griffon vultures. *Journal of Experimental Biology*, *215*(6), 986–996. https://doi.org/10.1242/jeb.058602

Patterson, A., Gilchrist, H. G., Chivers, L., Hatch, S., & Elliott, K. (2019). A comparison of techniques for classifying behavior from accelerometers for two species of seabird. *Ecology and Evolution*, *9*(6), 3030–3045. https://doi.org/10.1002/ece3.4740

Shamoun-Baranes, J., Bom, R., Loon, E. E. van, Ens, B. J., Oosterbeek, K., & Bouten, W. (2012). From Sensor Data to Animal Behaviour: An Oystercatcher Example. *PLOS ONE*, *7*(5), e37997. https://doi.org/10.1371/journal.pone.0037997
